# P-Rex1 is dispensable for Erk activation and mitogenesis in breast cancer

**DOI:** 10.18632/oncotarget.25584

**Published:** 2018-06-19

**Authors:** Laura Barrio-Real, Cynthia Lopez-Haber, Victoria Casado-Medrano, Alexander G. Goglia, Jared E. Toettcher, Maria J. Caloca, Marcelo G. Kazanietz

**Affiliations:** ^1^ Department of Systems Pharmacology and Translational Therapeutics, Perelman School of Medicine, University of Pennsylvania, Philadelphia, PA 19104-6160, USA; ^2^ Department of Molecular Biology, Princeton University, Princeton, NJ 08544-1014, USA; ^3^ Instituto de Biología y Genética Molecular, Consejo Superior de Investigaciones Científicas, Universidad de Valladolid, 47003 Valladolid, Spain

**Keywords:** P-Rex1, Rac1, Erk, Akt, mitogenesis

## Abstract

Phosphatidylinositol-3,4,5-Trisphosphate Dependent Rac Exchange Factor 1 (P-Rex1) is a key mediator of growth factor-induced activation of Rac1, a small GTP-binding protein widely implicated in actin cytoskeleton reorganization. This Guanine nucleotide Exchange Factor (GEF) is overexpressed in human luminal breast cancer, and its expression associates with disease progression, metastatic dissemination and poor outcome. Despite the established contribution of P-Rex1 to Rac activation and cell locomotion, whether this Rac-GEF has any relevant role in mitogenesis has been a subject of controversy. To tackle the discrepancies among various reports, we carried out an exhaustive analysis of the potential involvement of P-Rex1 on the activation of the mitogenic Erk pathway. Using a range of luminal breast cancer cellular models, we unequivocally showed that silencing P-Rex1 (transiently, stably, using multiple siRNA sequences) had no effect on the phospho-Erk response upon stimulation with growth factors (EGF, heregulin, IGF-I) or a GPCR ligand (SDF-1). The lack of involvement of P-Rex1 in Erk activation was confirmed at the single cell level using a fluorescent biosensor of Erk kinase activity. Depletion of P-Rex1 from breast cancer cells failed to affect cell cycle progression, cyclin D1 induction, Akt activation and apoptotic responses. In addition, mammary-specific P-Rex1 transgenic mice (MMTV-P-Rex1) did not show any obvious hyperproliferative phenotype. Therefore, despite its crucial role in Rac1 activation and cell motility, P-Rex1 is dispensable for mitogenic or survival responses in breast cancer cells.

## INTRODUCTION

Rac small G-proteins (Rac1, Rac2, Rac3 and RhoG) play essential roles in the regulation of actin cytoskeleton dynamics and cell motility, and control other cellular functions such as adhesion, polarization, survival, cell cycle progression and gene expression. Like most Rho GTPases, Rac proteins act as molecular switches that cycle between GDP-bound (inactive) and GTP-bound (active) states. Guanine nucleotide Exchange Factors (GEFs) promote GTP loading onto Rac, leading to the activation of a number of downstream effectors, such as Pak kinases, which drive the Rac phenotypes. Rac activity is turned off by GTPase Activating Proteins (GAPs) that accelerate its intrinsic rate of GTP hydrolysis. Once in the inactive conformation, Guanine nucleotide-Dissociation Inhibitors (GDIs) bind to and stabilize Rac, and preclude it from getting activated [1–3]. It has been well established over the last two decades that Rac and their regulators play fundamental roles in cancer progression [3–7]. Changes in abundance or mutational status of Rac and their regulators, contribute to tumor growth and metastatic dissemination of cancer cells [3, 8–11].

Previous studies identified the Rac-specific GEF Phosphatidylinositol-3,4,5-Trisphosphate Dependent Rac Exchange Factor 1 (P-Rex1) as a main player in Rac activation [12–14]. P-Rex1 is dually activated by phosphatidylinositol (3,4,5)-trisphosphate (PIP3), a PI3K product, and Gβγ subunits released upon stimulation of G-protein-coupled receptors (GPCRs). As a member of the Dbl family of Rho-GEFs, P-Rex1 has a characteristic DH (Dbl homology) domain responsible for the GDP/GTP exchange activity and an adjacent PH (pleckstrin homology) domain. DEP (dishevelled, Egl-10 and pleckstrin), PDZ (postsynaptic density 95, discs large, zonula occludens-1) and IP4P (inositol polyphosphate 4-phosphatase)-related domains present in P-Rex1 have been less characterized [12, 13, 15]. Although originally identified in neutrophils [13, 16, 17], it was subsequently reported that P-Rex1 is highly expressed in tumors, most notably in breast cancer [18, 19]. P-Rex1 is essentially not detectable in normal human mammary tissue, however it is prominently overexpressed in breast tumors of luminal subtype. Its expression has been linked to a high probability of metastasis and overall reduced survival in patients. Consistent with results in human specimens, P-Rex1 up-regulation is observed in luminal breast cancer cell lines such as MCF-7, T-47D, and BT-474, whereas it is essentially undetectable in “normal” MCF-10 mammary cells or basal/triple negative cell lines such as MDA-MB-231 cells [18]. P-Rex1 up-regulation in luminal breast cancer has been associated with deregulated epigenetic mechanisms that lead to *PREX1* gene promoter demethylation [20]. P-Rex1 expression has been also reported in prostate cancer, ovarian cancer, melanoma, and glioblastoma [21–24].

Studies have clearly established a fundamental role for P-Rex1 in breast cancer cell motility. Indeed, silencing P-Rex1 expression from luminal breast cancer cells severely affects Rac1 activation, actin cytoskeleton reorganization, ruffle/lamellipodia formation, and migration in response to growth factor stimulation [18]. Mechanistically, P-Rex1 integrates signals converging from tyrosine-kinase receptors and GPCRs (such as CXCR4), reflecting the dual PI3K- and Gβγ requirement for its activation [14, 18, 25, 26]. More recently, a mechanistic link between PI3K and MEK/Erk via P-Rex1/Rac1/c-Raf has been reported in breast cancer cells. According to these studies, silencing P-Rex1 expression from breast cancer cells impairs the activation of Erk, a major driver of mitogenic signaling, thus arguing for the involvement of this Rac-GEF in breast cancer cell proliferation [27–29]. This was unforeseen considering that P-Rex1 knockdown in melanoma cells did not affect Erk phosphorylation status or proliferation, and P-Rex1 overexpression had no effect on the tumorigenic activity of prostate cancer cells [21, 30]. In view of these discrepancies, and taking into consideration the prominent role of P-Rex1 in human breast cancer progression, here we sought to thoroughly examine whether P-Rex1 is or is not implicated in Erk mitogenic activity in breast cancer cells. Our analysis unambiguously shows that P-Rex1 is dispensable for the activation of Erk. In addition, P-Rex1 is not involved in Akt activation and resistance to cell death stimuli in breast cancer cell models.

## RESULTS

### P-Rex1 is required for Rac1 activation and breast cancer cell motility, but not for Erk activation

**Figure 1:**
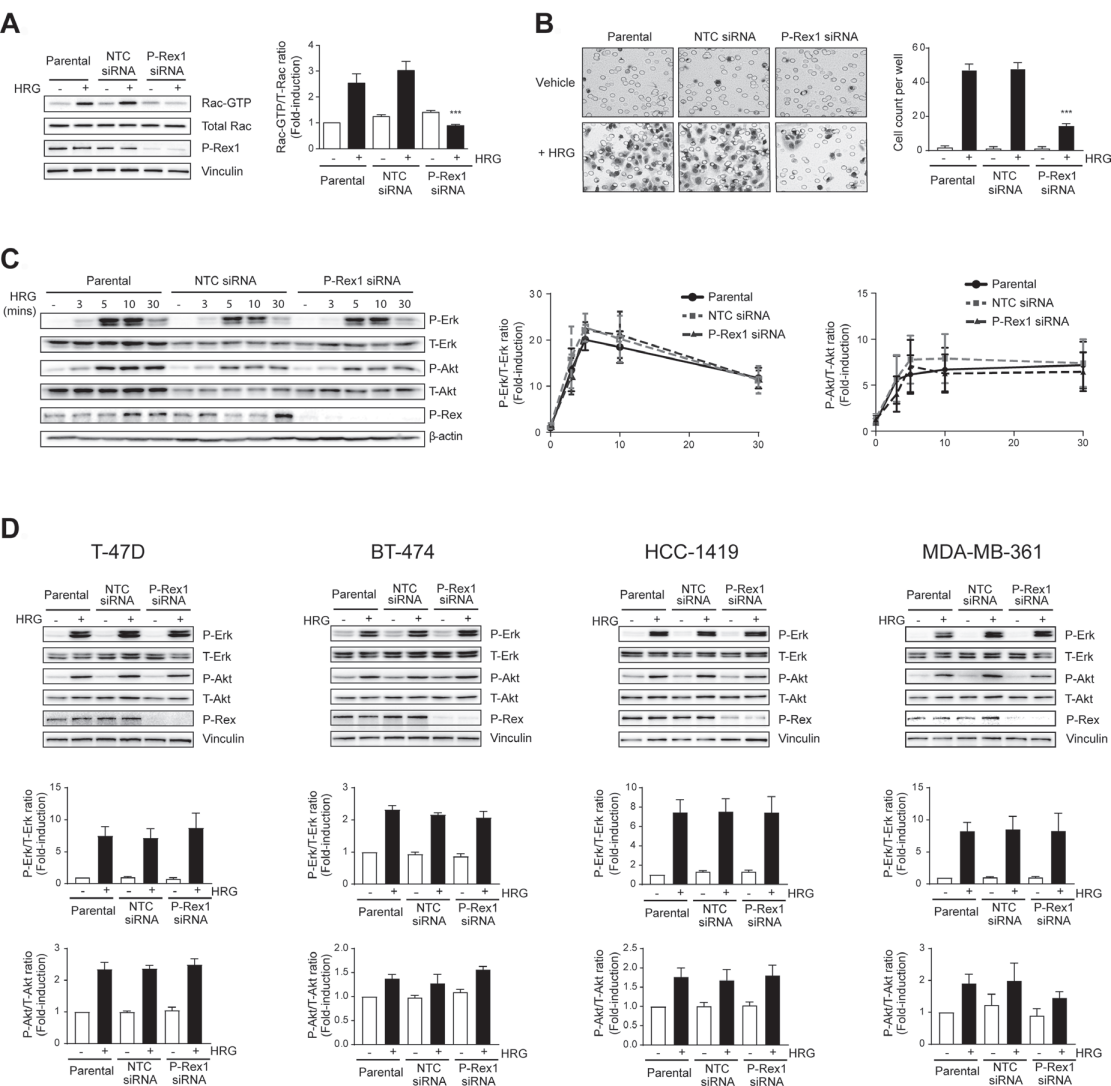
P-Rex1 silencing abrogates Rac activation and migration by heregulin, but does not affect Erk or Akt activation in breast cancer cells Cells were transfected with P-Rex1 siRNA (pool) or non-target control (NTC) siRNA. After 16 h, cells were serum starved for 24 h and subject to either HRG or vehicle treatment. (**A**) Rac1-GTP levels in MCF-7 cells in response HRG stimulation (20 ng/ml, 5 min) were determined using a pull-down assay. *Left panel*, representative experiment. *Right panel*, densitometric analysis of Rac-GTP, normalized to the total Rac1 levels. (**B**) Migration of MCF-7 cells in response to HRG (20 ng/ml, 16 h), as determined using a Boyden chamber assay. *Left panel*, representative experiment. *Right panel*, quantification of 3 independent experiments. (**C**) Time-course activation of Erk and Akt in MCF-7 cells in response to HRG (20 ng/ml). *Left panel*, representative experiment. *Middle and right panels*, densitometric analysis of phospho-Erk and phospho-Akt levels, normalized to the total Erk or Akt levels, respectively. (**D**) Activation of Erk and Akt in T-47D, BT-474, HCC-1419, and MDA-MB-361 by HRG (20 ng/ml. 5 min) subject to P-Rex1 or NTC siRNA. *Upper panels,* representative experiments. *Middle and lower panels*, densitometric analysis of phospho-Erk and phospho-Akt levels normalized to total levels. For all panels, results were expressed as fold-change relative to parental cells with vehicle stimulation. Data were expressed as mean ± S.E.M. of 3 independent experiments. ^***^p < 0.001 *vs.* parental.

We have previously reported that P-Rex1, which is highly expressed in luminal breast cancer cells, is the main Rac-GEF mediating Rac1 activation in response to ErbB receptor ligands [18]. A question we then wished to address is whether P-Rex1 plays any role in mitogenic signaling in breast cancer cells. Towards this end, we silenced P-Rex1 expression from MCF-7 breast cancer cells using a siRNA duplex pool, as done previously [18, 31]. When Rac1-GTP levels in response to the ErbB3 receptor ligand HRG were measured using a PBD-pull-down assay, a substantial reduction in Rac1 activation was observed in P-Rex1 knocked-down cells compared to parental MCF-7 cells or cells transfected with a non-target control (NTC) siRNA duplex pool (Figure 1A). Likewise, P-Rex1 siRNA also impaired MCF-7 cell motility, as determined using a Boyden chamber (Figure 1B), therefore confirming the expected functional consequences of P-Rex1 silencing [18]. To determine if P-Rex1 is implicated in Erk activation by HRG, we first carried out a time-course analysis. This experiment revealed no temporal differences or changes in the maximum Erk activation response (which occurs at 5 min) as a consequence of P-Rex1 silencing. A similar kinetics analysis for the activation of Akt also revealed P-Rex1-independent activation of this pro-survival kinase (Figure 1C). To determine if these results could be extended to other luminal breast cancer cells that express high P-Rex1 levels, we carried out similar experiments in T-47D, BT-474, HCC-1419, and MDA-MB361 cells. We previously reported that P-Rex1 siRNA impairs Rac1 activation by HRG in all these cell lines [18]. Figure 1D shows that HRG strongly activated Erk or Akt in these cell lines, but in no case were these responses changed as a consequence of P-Rex1 siRNA depletion.

**Figure 2:**
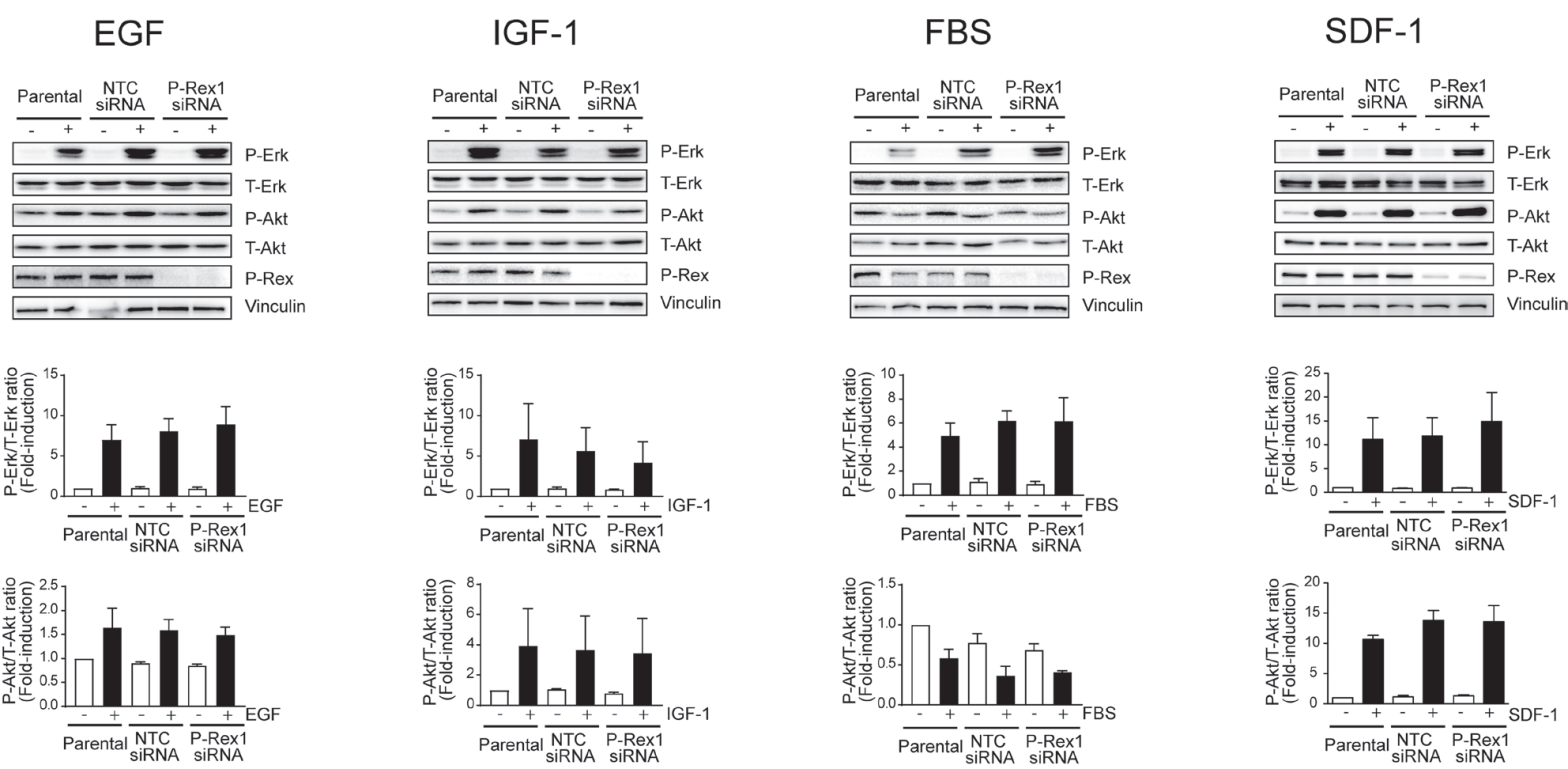
P-Rex1 is not involved in Erk and Akt activation by multiple growth factors and SDF-1 MCF-7 cells were transfected with P-Rex1 siRNA (pool) or non-target control (NTC) siRNA. After 16 h, cells were serum starved for 24 h and stimulated with EGF (100 ng/ml, 2 min), IGF-I (100 ng/ml, 5 min), FBS (10%, 10 min), or SDF-1 (100 ng/ml, 5 min). *Upper panels*, representative experiments. *Middle and lower panels*, densitometric analysis of phospho-Erk and phospho-Akt levels normalized to total levels. Results were expressed as fold-change relative to parental cells with vehicle stimulation. Data were expressed as mean ± S.E.M. of 3 independent experiments. No statistically significant differences were observed between parental, NTC and P-Rex1-depleted cells.

Next, we analyzed the effect of knocking down P-Rex1 on Erk activation by different growth factors. MCF-7 cells were stimulated with EGF and IGF-I, which activate Rac1 in a P-Rex1-dependent manner [18], as well as with 10% FBS. In all cases, significant Erk activation was observed. However, silencing P-Rex1 caused essentially no changes in the elevation of phospho-Erk levels by EGF, IGF-I, or FBS, relative to parental cells or cells subjected to NTC siRNA. Assessment of Akt activation revealed a 2–4-fold activation by the different growth factors, although FBS caused a slight reduction in phospho-Akt levels. Regardless, changes in Akt activation by the different stimuli were similar in parental, NTC, and P-Rex1 siRNA transfected cells (Figure 2; time-course in Supplementary Figure 1). To confirm these results, we used a stable depletion approach using two different P-Rex1 shRNA lentiviruses. When cells stably depleted of P-Rex1 cells were challenged with different stimuli (HRG, EGF, IGF-I, FBS), we found that the elevation in phospho-Erk and phospho-Akt levels were similar than those in cells infected with a NTC shRNA lentivirus. A representative experiment using two P-Rex1 shRNA lentiviruses is shown in Supplementary Figure 2A.

It is well established that stimulation of Gi-coupled receptors leads to P-Rex1/Rac activation. For example, the CXCR4 ligand SDF-1 causes a significant activation of Rac1 and cell motility in breast cancer cells that depends on P-Rex1 [18, 31]. Nevertheless, activation of Erk and Akt by SDF-1 remained basically unchanged upon silencing the expression of P-Rex1 from breast cancer cells (Figure 2; time-course in Supplementary Figure 1).

### Assessment of Erk activation dynamics in single cells

To further assess a potential dependence of Erk activation via P-Rex1, we used a fluorescent biosensor of Erk kinase activity to measure the efficiency of Erk activation in living single cells. This approach uses an Erk kinase translocation reporter (KTR) bound to a fluorescent protein that undergoes nuclear export in response to stimuli upon Erk-mediated phosphorylation. The system enables dynamic visualization of Erk signaling in living cells that can be determined at a single cell level, and allows assessment of both population-level and cell-to-cell variability [32–34].

**Figure 3:**
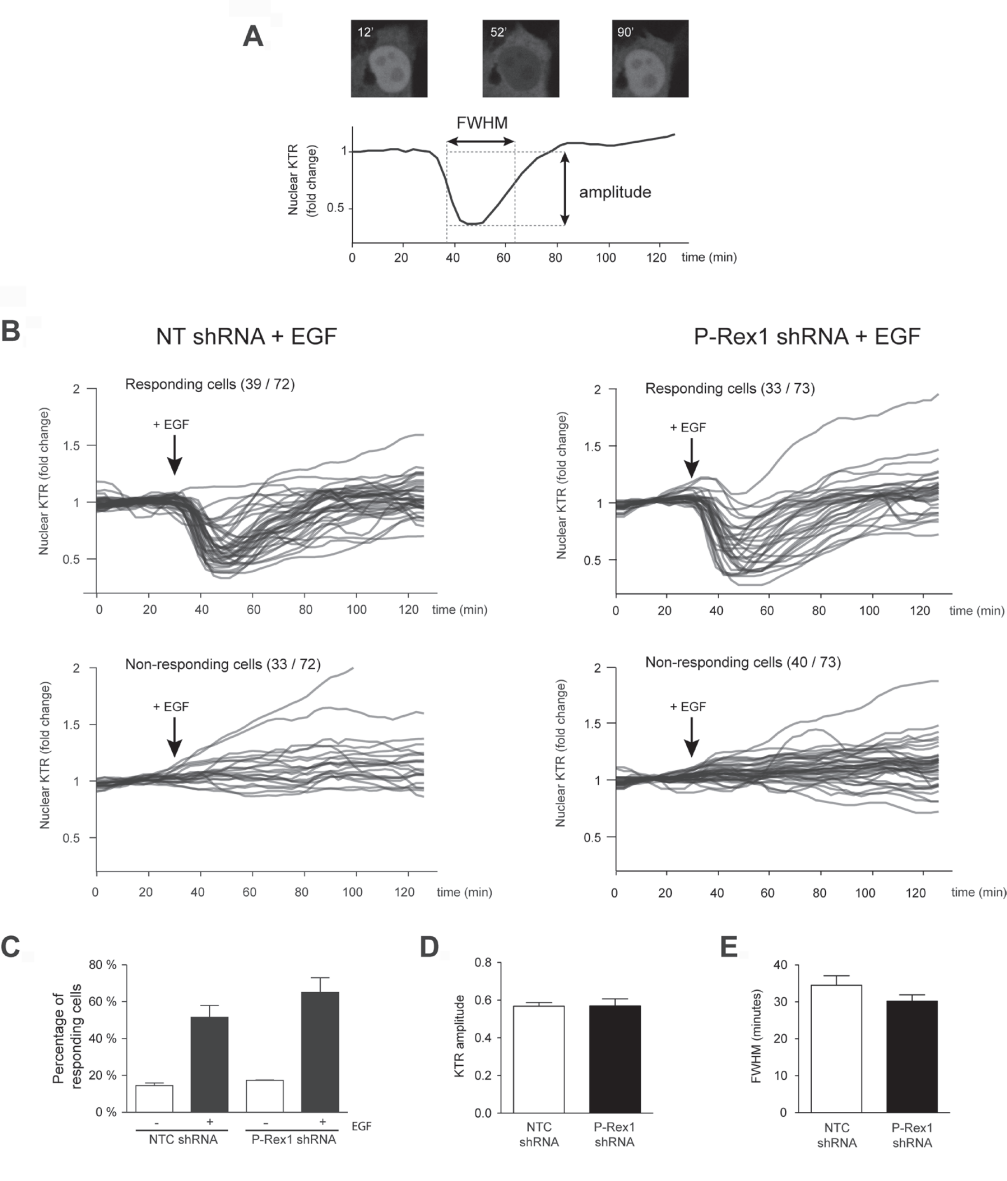
Assessment of Erk activation in single MCF-7 cells using the Erk-KTR (**A**) A representative single-cell trace of nuclear Erk-KTR dynamics in MCF-7 cells over 2.5 h is shown, with raw images of the individual cell depicted at 12 min (before 100 ng/ml EGF addition), 52 min (22 min after EGF addition), and 90 min (60 min after EGF addition). The maximum amplitude of the KTR response corresponds to the degree to which Erk becomes activated. The full-width at half maximum (FWHM) represents the width of the Erk activity pulse (*i.e.*, the duration of Erk response). (**B**) Single-cell traces of Erk-KTR response to EGF in MCF-7 cell populations subject to NTC or P-Rex1 shRNA. Cells were imaged every 3 min over the course of ∼2.5 h. All traces for a given condition are taken from a single well, and are plotted as fold-change relative to the first ten (non-stimulated) time points. (**C**) The number of responding and non-responding cells was similar in both cell populations. (**D**) KTR amplitude in response to EGF. (**E**) FWHM of KTR response to EGF. For (A–C), at least 100 cells were analyzed in each condition. Results are expressed as mean ± S.E.M of 4 independent experiments.

MCF-7 cells, either subject to NTC shRNA or P-Rex1 shRNA, were infected with the Erk-KTR lentivirus (as described in Experimental Procedures), serum starved and subject to EGF stimulation. Figure 3A shows a representative single-cell trace of nuclear Erk-KTR dynamics in MCF-7 cells subject to EGF treatment. Comparison of Erk-KTR dynamics between control and P-Rex-1-depleted cells is presented in Figure 3B, which shows a similar pattern in both cell populations (Figure 3C). A quantitative analysis of the amplitude of the Erk-KTR response, that corresponds to the degree to which Erk becomes activated (Figure 3D), and the full-width at half-maximum (FWHM), which represents the width of the Erk activity pulse (*i.e.*, the duration of Erk response) (Figure 3E), revealed essentially similar results between control and P-Rex1-depleted MCF-7 cells. Therefore, in agreement with conclusions from Western blot experiments, the dynamics of Erk activation in living cells is not affected by P-Rex1 silencing, thus supporting the lack of involvement of this Rac-GEF in Erk activation.

### P-Rex1 is not required for cell cycle progression and survival

**Figure 4:**
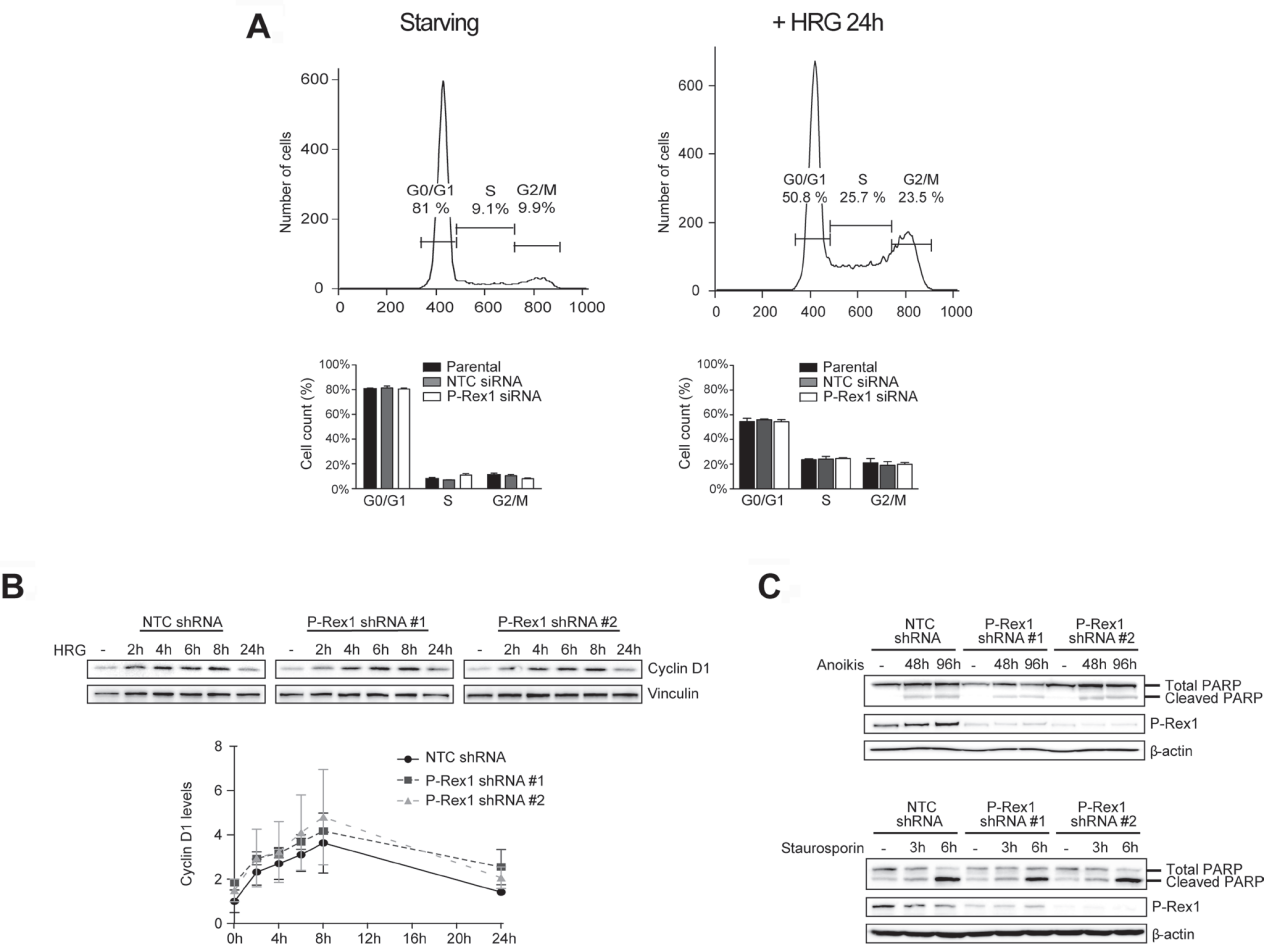
Analysis of P-Rex1 depletion effects on cell cycle progression and apoptosis (**A**) MCF-7 cells subject to P-Rex1 siRNA (pool) or non-target control (NTC) siRNA were serum starved for 24 h, stimulated with HRG (20 ng/ml) or vehicle for 24 h, and subject to cell cycle analysis by FACS. *Upper panels*, representative histograms. *Lower panels*, distribution of cells in the different phases of cell cycle. Data are expressed as mean ± S.E.M. of 3 independent experiments. (**B**) Cyclin D1 induction in response to HRG (20 ng/ml, 0-24 h) in MCF-7 cells subject to either P-Rex1 or NTC shRNA or two different P-Rex1 shRNAs as determined by Western blot. *Upper panel*, representative experiment. *Lower panel*, densitometric analysis. Results were expressed as fold-change relative to parental cells with vehicle stimulation. Data were expressed as mean ± S.E.M. of 3 independent experiments. (**C**) PARP cleavage in response to cell detachment (anoikis) or staurosporin. *Upper pan*el, MCF-7 cells subject to either NTC shRNA or two differents P-Rex1 shRNAs, were growth in suspension for 48 or 96 h. *Lower panel*, MCF-7 cells were treated with staurosporin (1 µM, 3–6 h). A representative experiment is shown. Similar effects were found in 2 additional experiments.

The Erk pathway plays a central role in cell cycle progression and proliferation [35–38]. A report suggested that P-Rex1 controls the induction of cyclin D1 in breast cancer cells [29]. As we were unable to find any involvement of P-Rex1 in Erk activation in breast cancer cells, we asked if silencing P-Rex1 has any impact on cell cycle progression. FACS analysis revealed that the pattern of distribution of cells in G1/S, M and G2 phases in response to HRG stimulation is similar in parental, NTC and P-Rex-1-depleted MCF-7 cells, either transiently or stably (Figure 4A and Supplementary Figure 2B). Consistent with these results, there were no changes in the kinetics or magnitude of cyclin D1 induction by HRG (Figure 4B).

In order to assess if P-Rex1 has any role in resistance to apoptosis, we examined the effect of P-Rex1 depletion on anoikis, a form of programmed cell death that occurs in anchorage-dependent cells when they detach from the surrounding extracellular matrix [39]. MCF-7 cells were plated at low confluence in poly-HEMA coated plates to prevent cell attachment to the plastic, and detached cells were collected 48 h or 96 h later. Figure 4C shows that PARP cleavage, a readout of apoptosis, was similar in control and P-Rex1-depleted cells. Similar results were observed when we treated cells with the apoptotic agent staurosporin.

### Mammary-specific P-Rex1 mice does not show a hyperproliferative phenotype

**Figure 5:**
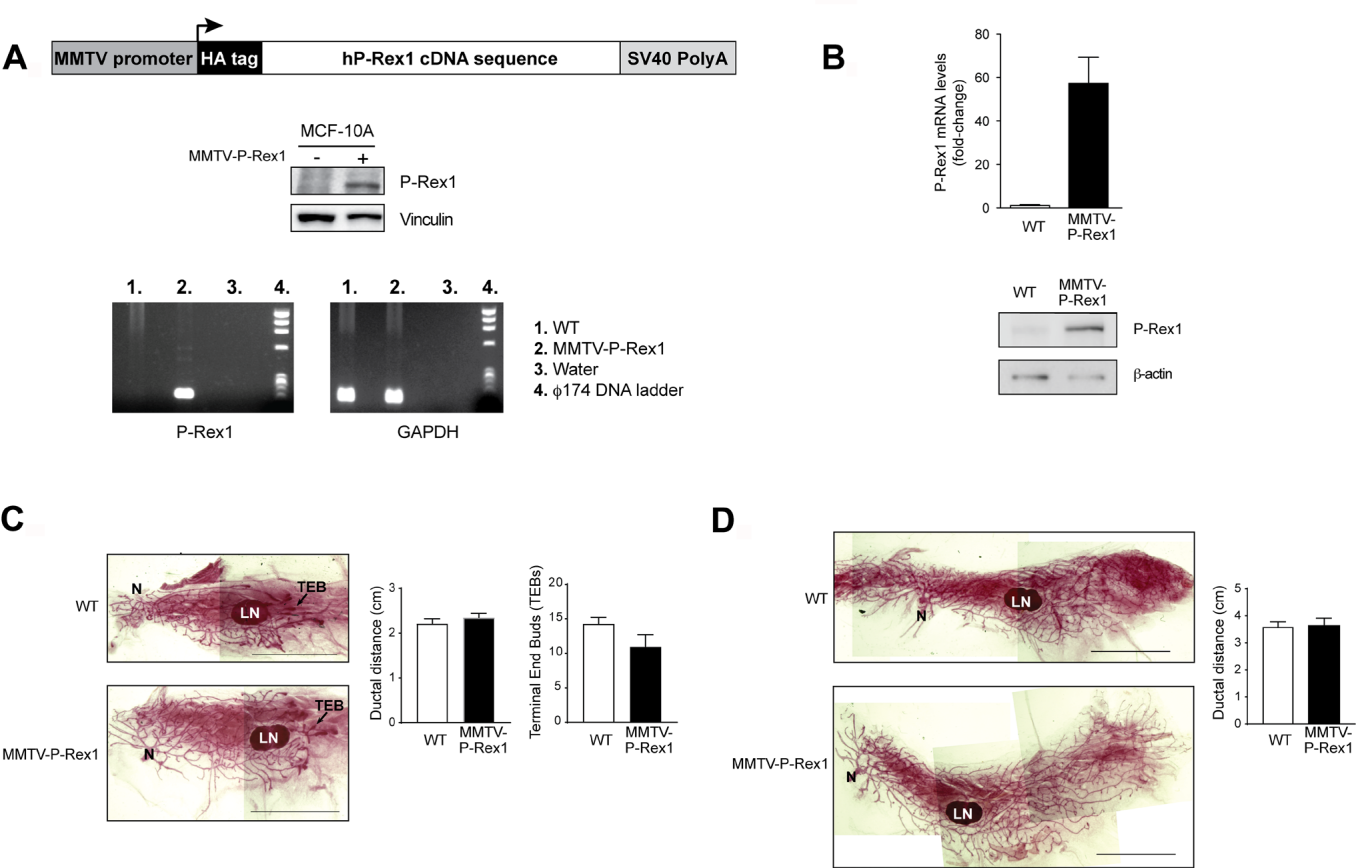
Characterization of MMTV-P-Rex1 mice (**A**) *Upper panel*, schematic representation of the P-Rex1 transgene construct; *middle panel*, expression of the construction in transfected MCF-10A cells; *lower panel*, representative PCR genotyping. (**B**) P-Rex1 expression in primary cultures from murine mammary glands. *Upper panel*, representative qPCR using primers designed to specifically amplify the human P-Rex1 transgene. *Lower panel*, representative Western blot analysis of transgene expression. (**C** and **D**) Analysis of mammary glands from 6-week old (C) or 12-week old (in pro-estrus phase) (D). *Left panels*, representative whole mount of inguinal murine mammary glands of MMTV-P-Rex1 and wild-type mice. *Right panels*, graphic representation of the ductal distance (for 6-month and 12-month old) and number of terminal end buds (TEBs) per mammary gland (for 6-week old). Results are expressed as mean ± S.E.M. (*n* = 3). *N*, nipple; *LN*, lymph node. *Scale bar*: 1 cm.

P-Rex1 is overexpressed in luminal breast cancer, however its expression is negligible in normal human mammary glands [18]. Similarly, we were unable to detect P-Rex1 expression in murine mammary glands (data not shown). In order to determine if expression of P-Rex1 affects the proliferation of normal mammary glands, we generated a tissue-specific transgenic P-Rex1 mouse model, MMTV-P-Rex1. A schematic representation of the transgenic construct, expression upon transfection into MCF-10A cells, and genotyping of transgenic mice are shown in Figure 5A. Expression of the *PREX1* transgene was confirmed by qPCR and Western blot in primary mammary epithelial cells derived from the P-Rex1 transgenic mice (Figure 5B).

P-Rex1 transgenic female mice nursed their offspring to maturity and showed no gross defects on the mammary gland architecture, suggesting that P-Rex1 overexpression does not impair mammary gland function. Mammary glands of P-Rex1 transgenic and wild-type female sibling mice were collected at different stages of development (6-week old, 12-week old, involution, 13-month old nulliparous, and 13-month old multiparous). Visual inspection of the fixed mammary glands did not reveal any obvious pre-neoplastic lesions or any detectable morphological anomalies in any group (data not shown). The ductal distance between the origin of the mammary gland (nipple) and the distal end bud in 6-week old (puberty) and 12-week old (early adulthood) mice was similar in MMTV-P-Rex1 and wild-type mice. Likewise, no statistically significant differences in the number of terminal end buds (TEDs) could be found between control and MMTV-P-Rex1 mice (Figure 5C and 5D).

## DISCUSSION

The present study shed light on the controversial issue of P-Rex1 as a mediator of Erk activation in breast cancer cells. Our results unequivocally established that P-Rex1 is dispensable for Erk activation by stimulation of tyrosine-kinase receptors or GPCRs in breast cancer cells, and accordingly it is not implicated in mitogenic signaling. Due to the discrepancies found with other reports [27–29], we took a stringent and comprehensive experimental approach, which involved the use of multiple luminal breast cancer cell lines expressing P-Rex1, a range of stimuli that includes different growth factors and a GPCR ligand, transient and stable approaches to silence P-Rex1, and multiple siRNA sequences. Notwithstanding the different strategies used to assess the Erk response, it was not affected by P-Rex1 depletion. In all cases, P-Rex1 turned out to be dispensable for the activation of the survival kinase Akt, as we previously reported [18]. Under these conditions, there is essentially no Rac1 activation or motility in response to stimuli, therefore making it unlikely that residual P-Rex1 levels (5–30%, depending on the cell line) drives a full signaling response. Quite interestingly, mammary-specific expression of P-Rex1 in mice did not display any evident phenotype. A previous study showed that expression of an activated V12-Rac allele in mice under the control of the MMTV promoter leads to sustained Rac signaling activation, and generates a benign mammary gland phenotype characterized by a slight reduction in apoptosis, however lacking any signs of increased epithelial proliferation [40]. Together, this argues against the involvement of the P-Rex1/Rac signaling pathway in the control of mammary cell proliferation.

P-Rex1 was originally identified as the main GEF controlling Rac activation and Rac-mediated reactive oxygen species formation in neutrophils [13]. Years later, P-Rex1 was found to be highly expressed in a number of cancers, most notably in luminal breast cancer [18, 19]. Studies from our laboratory and others revealed that P-Rex1 is an essential mediator of Rac signaling activation in response to stimulation of tyrosine-kinase receptors and GPCRs in breast cancer cells, and that it integrates signals emanating from these receptors via PIP3 and Gβγ subunits, respectively [18, 19]. P-Rex1 redistributes to the plasma membrane in response to ErbB receptor activation in a P-Rex1-dependent manner, an effect that involves the transactivation of the Gi-coupled receptor CXCR4 [18]. ErbB receptor activation also up-regulates CXCR4 signaling via a transcriptional mechanism, thereby inducing the sensitization of the P-Rex1/Rac1 pathway to SDF-1 [26, 31]. As expected from its Rac-GEF activity, P-Rex1 plays fundamental roles in cell motility and invasiveness, and has been linked to metastatic dissemination of breast cancer cells [18]. Our present results, together with previous publications [18, 19, 31], support this conclusion. Moreover, P-Rex1 controls the expression of genes involved in invasiveness in breast cancer cells [41]. Similar effects of P-Rex1 in cell motility and invasion have been observed in other cancers, including prostate and ovarian cancer, melanoma, and glioblastoma [21–24]. The involvement of P-Rex1 in cancer cell migration *in vivo* has been demonstrated using a mouse model for melanoma metastasis (Lindsay *et al.*, Ref. 23).

The differences between our studies and previous reports regarding the involvement of P-Rex1 in Erk activation may be the consequence of multiple factors. One possibility is that P-Rex1 siRNA vectors or duplexes used in other studies are causing an “off-target” effect that impacts on Erk activity in a non-specific manner. We have transiently transfected a P-Rex1 siRNA duplexes pool, as well as generated cell lines with different P-Rex1 shRNA lentiviruses, and we consistently observed full Erk activation in response to stimuli. Liu *et al.* reported inhibitory effects on Erk activation by IGF-I and breast cell proliferation upon stable expression of two different P-Rex1 shRNAs, with no effects on Akt activation [29]. Also, ectopic overexpression of P-Rex1 in MDA-MB-231 cells, which do not express P-Rex1 [18], enhances Erk activation by IGF-I [29]. This approach may be questionable, since MDA-MB-231 is a basal/triple negative breast cancer cell line that does not express P-Rex1 endogenously, and in addition has multiple genetic alterations, including Ras mutations [42], which could impact mitogenic responses. It is interesting that the expression of P-Rex1 in breast cancer cells correlates with the sensitivity to PI3K inhibition [27, 28], however this does not rule out other PI3K-dependent mediators. The PI3K/Rac/Erk-dependent induction of cyclin D1 previously observed in breast cancer cells may involve P-Rex1-independent pathways that are mediated by the NF-κB pathway [43].

It is important to mention that our results showing the lack of involvement of P-Rex1 in proliferation in breast cancer cells fit with those observed in other models. In a recent study, Cox, Der and others used three different siRNA duplexes to knock down P-Rex1 in four different melanoma cell lines. Their results clearly show that P-Rex1 silencing neither affected the proliferative capacity of melanoma cell lines nor regulated Erk phosphorylation, while still causing major inhibitory effects on Rac activation and invasive behavior. Rather, their findings revealed that in melanoma cells, Erk activity is required for maintaining the expression of P-Rex1 via transcriptional and post-transcriptional mechanisms [30]. A recent study also showed that P-Rex1 siRNA in glioblastoma cells did not affect growth, whereas it markedly affected motility and invasion [24]. These results align with ours in luminal breast cancer models, and support a specific role for P-Rex1 in cell motility rather than in cell growth. Along the same line, it was shown that expression of P-Rex1 did not affect primary tumor growth of prostate cancer cells in xenograft models [21], whereas it is required for melanoma cell metastatic dissemination *in vivo* (Lindsay *et al.*, Ref. 23). The absence of an evident phenotype in the mouse mammary gland of P-Rex1-transgenic mice supports the concept that P-Rex1 does not play any major role in mammary epithelial cell proliferation.

In summary, our study shed light into the controversial issue of P-Rex1 as a regulator of mitogenic signaling. Our results clearly demonstrate that P-Rex1 is not involved in the activation of the Erk mitogenic pathway and consequently does not play a significant role in the growth of breast cancer cells. The most prominent role for P-Rex1 in breast cancer cells is the control of motility signaling via Rac1, a crucial step for metastatic dissemination. Thus, this study contributes to a better understanding of the functional properties of this important Rac-GEF.

## MATERIALS AND METHODS

### Cell lines and reagents

Authenticated breast cells lines were purchased from the ATCC (Manassas, VA, USA). MCF-7 cells were cultured in RPMI supplemented with 10% FBS, 2 mM glutamine, 1 mM sodium pyruvate, 10 μg/ml insulin, 100 U/ml penicillin, and 100 μg/ml streptomycin. T-47D, BT-474, HCC-1419 and MDA-MB-361 cells were cultured in DMEM supplemented with 10% FBS, 100 U/ml penicillin, and 100 μg/ml streptomycin. MCF-10A were cultured in DMEM–F-12 medium supplemented with 5% horse serum, 20 ng/ml EGF, 0.5 μg/ml hydrocortisone, 100 ng/ml cholera toxin, 10 μg/ml insulin, 100 U/ml penicillin, and 100 μg/ml streptomycin. Heregulin β1 (HRG), IGF-1, EGF and SDF-1 were purchased from R&D Systems (Minneapolis, MN, USA).

### Erk and Akt activation by Western blot

Erk and Akt activation in response to stimuli was determined by Western blot using anti-phospho-Erk1/2 (Thr202/Tyr204) and anti-phospho-Akt (Ser473) antibodies (Cell Signaling Technology, Beverly, MA, USA). Anti-Erk1/2 and anti-Akt antibodies (Cell Signaling Technology) were used for detection of total Erk1/2 and Akt, respectively. Anti-β-actin (BD Biosciences, Franklin Lakes, NJ, USA) and anti-vinculin antibodies (Sigma-Aldrich, St. Louis, MO, USA) were used for loading controls.

Western blots were done essentially as previously described [31]. Briefly, cells were lysed in a buffer containing 2% SDS, 62.5 mM Tris-HCl, pH 6.8, 10% glycerol, 5% 2-mercaptoethanol, and 0.002% bromophenol blue, and extracts were subjected to SDS-polyacrylamide gel electrophoresis (PAGE). Bands were visualized by enhanced chemiluminescence (ECL). Images were captured using an Odyssey Fc system (Li-Cor Biosciences; Lincoln, NE, USA). Image processing and densitometry analysis were carried out using the Image Studio Lite software (Li-Cor Biosciences).

### siRNA and generation of P-Rex1-depleted stable cell lines

For transient depletion of P-Rex1, we used an ON-TARGETplus siRNA pool from Dharmacon (Lafayette, CO, USA). ON-TARGETplus non-targeting pool was used as a control. Small interfering RNAs (siRNAs) were transfected with Lipofectamine RNAi/Max (Invitrogen-Life Technologies, Grand Island, NY, USA).

For stable depletion of P-Rex1, cells were infected with control shRNA or P-Rex1 shRNA Mission^®^ lentiviral transduction particles from Sigma-Aldrich (Cat. # SHVRSNM_020820) according to the manufacturer’s protocol, and pools were selected with puromycin (0.5 µg/ml). Two different shRNA sequences were used (TRCN0000044794 [P-Rex1 shRNA#1] and TRCN0000418541 [P-Rex1 shRNA#2]. Expression of P-Rex1 in cell lines was detected using an anti-P-Rex1 antibody (Sigma-Aldrich).

### Rac1-GTP pull-down assays

After serum starvation for 24 h, cells were stimulated with HRG (20 ng/ml) for 5 min. Rac1-GTP levels were determined with a pull-down assay using the p21-binding domain (PBD) of Pak1, as described previously [44]. Briefly, cells were lysed in a pull-down buffer containing 20 mM Tris-HCl, pH 7.4, 150 μM NaCl, 5 mM MgCl2, 0.5% NP-40, 5 mM β-glycerophosphate, 1 mM DTT, protease inhibitors, and 10 μg/ml GST-PBD. Lysates were cleared by centrifugation (10 min at 4° C, 13,000 × g) and incubated with glutathione-Sepharose 4B beads (GE Healthcare, Mickleton, NJ, USA) for 45 min at 4° C. After centrifugation, beads were washed twice with the pull-down buffer and run on SDS-PAGE gels. Rac1-GTP was detected by Western blotting using an anti-Rac1 antibody (clone 23A8, Upstate Biotechnology, Lake Placid, NY, USA).

### Cell migration assay

After 24 h of serum starvation, cells were harvested with 1 mM EDTA and resuspended in 0.1% BSA-RPMI medium. Cells (3 × 10^4^ cells/well) were seeded in the upper compartment of a Boyden chamber (NeuroProbe, Gaithersburg, MD). A 12-μm-pore-size polycarbonate filter (NeuroProbe) coated overnight with type IV collagen in cold phosphate-buffered saline (PBS) was used to separate the upper and lower compartments. In the lower chamber, 0.1% BSA-RPMI with or without HRG (20 ng/ml) was used. After 16 h of incubation at 37° C, the non-migrating cells on the upper side of the membrane were wiped off the surface. Migrating cells on the lower side of the membrane were fixed, stained with Diff Quik stain set (Dade Behring-Siemens, Malvern, PA, USA), and counted by contrast microscopy in 5 independent fields.

### Cell cycle analysis and apoptosis assays

For cell cycle analysis, cells were serum-starved for 24 h and stimulated with HRG (20 ng/ml) for other 24 h. Cells were then harvested with 1 mM EDTA, washed with PBS, and fixed with ice-cold 100% ethanol. After washing again with PBS, cells were resuspended in Propidium Iodide (PI)/RNase Staining Solution (Cell Signaling Technology). Samples were analyzed on a BD FACSCalibur (BD Biosciences) and data were examined with FlowJo V10 software. Cyclin D1 antibody was detected by Western blot using an antibody from BD Biosciences.

For induction of apoptosis by anoikis, cell culture plates were coated with 6 mg/ml poly-HEMA (Sigma-Aldrich) in 95% ethanol and incubated at 37°C for several hours until they dry, and then washed with PBS. For single cell isolation, cells in suspension were passed through a 40 µm cell strainer (BD Biosciences) that retained the clumped cells, and then grown at low confluency (1 × 10^4^ cells/ml) to avoid cell-cell contact. Apoptosis was also induced by staurosporine (1 µM, 3–6 h, Enzo Life Science, Farmingdale, NY, USA). Total and cleaved PARP were detected by Western blot using an antibody from Cell Signaling Technology.

### Single cell Erk activation measurements

Production of lentiviruses for single cell signaling dynamics assays has been previously described [32–34]. Briefly, HEK 293T LX cells were plated in 6-well dishes at ∼40% confluency and co-transfected with pHR Erk-KTR-iRFP-2A-H2B-tagRFP expression plasmid and the requisite lentiviral helper plasmids (pCMV-dR8.91 and pMD2.G) using FuGENE HD transfection reagent (Promega, Madison, WI, USA). Lentiviral supernatant was harvested 2 days post-transfection, sterile filtered by passing through a 0.45 μm membrane, and supplemented to final concentrations of 5 μg/ml polybrene and 10 mM HEPES buffer. Imaging experiments were carried out in black-walled, 0.17 mm glass-bottom 96-well plates (Cellvis, Mountain View, CA, USA) pre-treated with bovine fibronectin (10 μg/ml in PBS). MCF-7 cells stably expressing either NTC shRNA or P-Rex1 shRNA were plated at low density (∼20%) and infected with 15 ml of Erk-KTR-2A-H2B lentivirus.

For live cell imaging of Erk KTR dynamics, 2 days after plating cells were serum starved in imaging medium (0% FBS, 20 mM HEPES) for 4 h. Fifty μl of mineral oil were added on top of the imaging medium in each well to prevent evaporation, and cells were maintained at 37° C and 5% CO_2_ throughout the experiment. Images were acquired every 3 min with a Nikon Eclipse Ti Spinning Disk Confocal Microscope (Nikon Instruments, Melville, NY, USA), using a 40× objective, a Prior linear motorized stage, and 650 nm (iRFP) and 561 (RFP) laser lines from an Agilent MLC 400 (Keysight Technologies, Santa Rosa, CA, USA). Acquisition was briefly paused after 30 min, and either DMSO vehicle control or a saturating dose of EGF (100 ng/ml) was added directly to cells on the microscope. Acquisition was then resumed and cells were imaged for an additional 2 h period. For quantification and analysis of live cell Erk KTR dynamics, we used MATLAB (The Mathworks, Natick, MA, USA) and ImageJ (NIH, Bethesda, MD, USA) software.

### Generation of MMTV-P-Rex1 transgenic mice and analysis of phenotype

In order to achieve mammary gland-specific expression of P-Rex1, the human P-Rex1 full-length cDNA was placed under the control of the MMTV promoter [45]. Transgenic mice were developed by pronuclear microinjection of FVB/NJ fertilized eggs in the Transgenic and Chimeric Mouse Facility at the University of Pennsylvania. Expression analysis of the transgene and genotyping of the transgenic mice were performed by PCR using the primers MMTV-P-Rex1-Forward (5′ TCT CCT CGG AGC TCT GCT AC) and MMTV-P-Rex1-Reverse (5′ GTT TTT GGC CAG AAT CTC CA). FVB/NJ inbred mice (used in maintenance of transgenic lines) were acquired from Jackson Laboratories (Bar Harbor, ME, USA). Mice were housed in individually ventilated cages on autoclaved hardwood bedding in an AAALAC accredited facility at University of Pennsylvania. All procedures were in compliance with the Public Health Service Guide for the Care and Use of Laboratory Animals.

Hemizygous P-Rex1-overexpressing (tg/0) and sibling wild-type (0/0) females on a pure FVB/NJ background were sacrificed at different ages: 6-week old, 12-week old in pro-estrus phase, involution (5 days post-weaning), 13-month old nulliparous and 13-month old multiparous mice (with two pregnancies). Murine estrus cycle was determined by vaginal cytological evaluation as previously described [46, 47]. Complete necropsy, macroscopic examination, tissue collection and processing were carried out as described in [48].

Whole inguinal mammary glands from experimental mice were dissected, mounted and spread in glass slides. The mounts were fixed in Carnoy’s fixative (ethanol, chloroform, glacial acetic acid. 6:3:1) for 4 h, and rehydrated. After staining with Carmine Red Stain for 16 h, samples were dehydrated and mounted with Permount (Fisher Scientific, Hampton, NH) in a glass cover slip. Whole mounts of mammary glands were photographed using a Nikon SMZ 1000 Stereo Microscope. Images were analyzed using ImageJ software. Mammary gland length was measured from the nipple area to the end of the longest duct (ductal distance), and terminal end buds (TEBs) were counted.

Mammary epithelial cells from experimental mice were isolated, and the primary cell culture expanded, as previously described [49]. Briefly, mammary tissue was mechanically dissociated, incubated in DMEM-F12 medium with 300 U/ml collagenase and 100 U/ml hyaluronidase (STEMCELL Technologies, Cambridge, MA) for 1 h at 37° C, and resuspended in 0.25% Trypsin-EDTA. Cells were maintained in DMEM-F12 containing 1 mM glutamine, 5 µg/ml insulin, 500 ng/ml hydrocortisone, and 10 ng/ml EGF, and 5% horse serum, and cultured at 37° C in 5% CO_2_.

Expression of P-Rex1 transgene expression was confirmed by qPCR and Western blot. For qPCR testing, total RNA from primary mammary epithelial cells was isolated using the RNeasy Mini Kit (Qiagen, Valencia, CA, USA), and reverse transcribed to cDNA with the TaqMan Reverse Transcription Kit (Invitrogen), as previously described [41]. qPCR was performed using predesigned sets of TaqMan primer/probes specific for human *PREX1* and the murine housekeeping gene *GAPDH* (used for normalization). Results were expressed as fold-change of the target gene by 2^−ΔΔCt^ and normalized to the wild-type murine sample. qPCR reaction was performed in triplicate.

### Statistical analysis

For statistical analysis of data, we used analysis of variance (ANOVA) and Bonferroni’s multiple-comparison test, using GraphPad Prism software (La Jolla, CA, USA).

## SUPPLEMENTARY MATERIALS FIGURES



## Abbreviations

ANOVA: analysis of variance
DEP domain: dishevelled, Egl-10 and pleckstrin domain
DMSO: dimethylsulfoxide
EGF: epidermal growth factor
FBS: fetal bovine serum
FWHM: full-width at half maximum
GEF: guanine nucleotide exchange factor
GPCR: G-protein-coupled receptor
IGF-I: insulin-like growth factor 1
IP4P: inositol polyphosphate 4-phosphatase domain
KTR: Erk kinase translocation reporter
MMTV: mouse mammary tumor virus
PDZ domain: postsynaptic density 95, discs large, zonula occludens-1 domain
PI: propidium iodide
PIP3: phosphatidylinositol (3:4:5)-trisphosphate
P-Rex1: Phosphatidylinositol-3:4:5-trisphosphate dependent Rac exchange factor 1
Q-PCR: quantitative polymerase chain reaction
RFP: red fluorescent protein
SDF-1: stromal cell-derived factor 1
siRNA: small interfering ribonucleic acid
TEB: terminal end buds
