# Supplementary material for: P-Rex1 is dispensable for Erk activation and mitogenesis in breast cancer
Source: Oncotarget. 2018 Jun 19;9(47):28612–24. doi: 10.18632/oncotarget.25584 (PMC6033363; doi:10.18632/oncotarget.25584)
Supplement: Supplementary file 1 [file oncotarget-09-28612-s001.pdf]

P-Rex1 is dispensable for Erk activation and mitogenesis in breast cancer

SUPPLEMENTARY MATERIALS

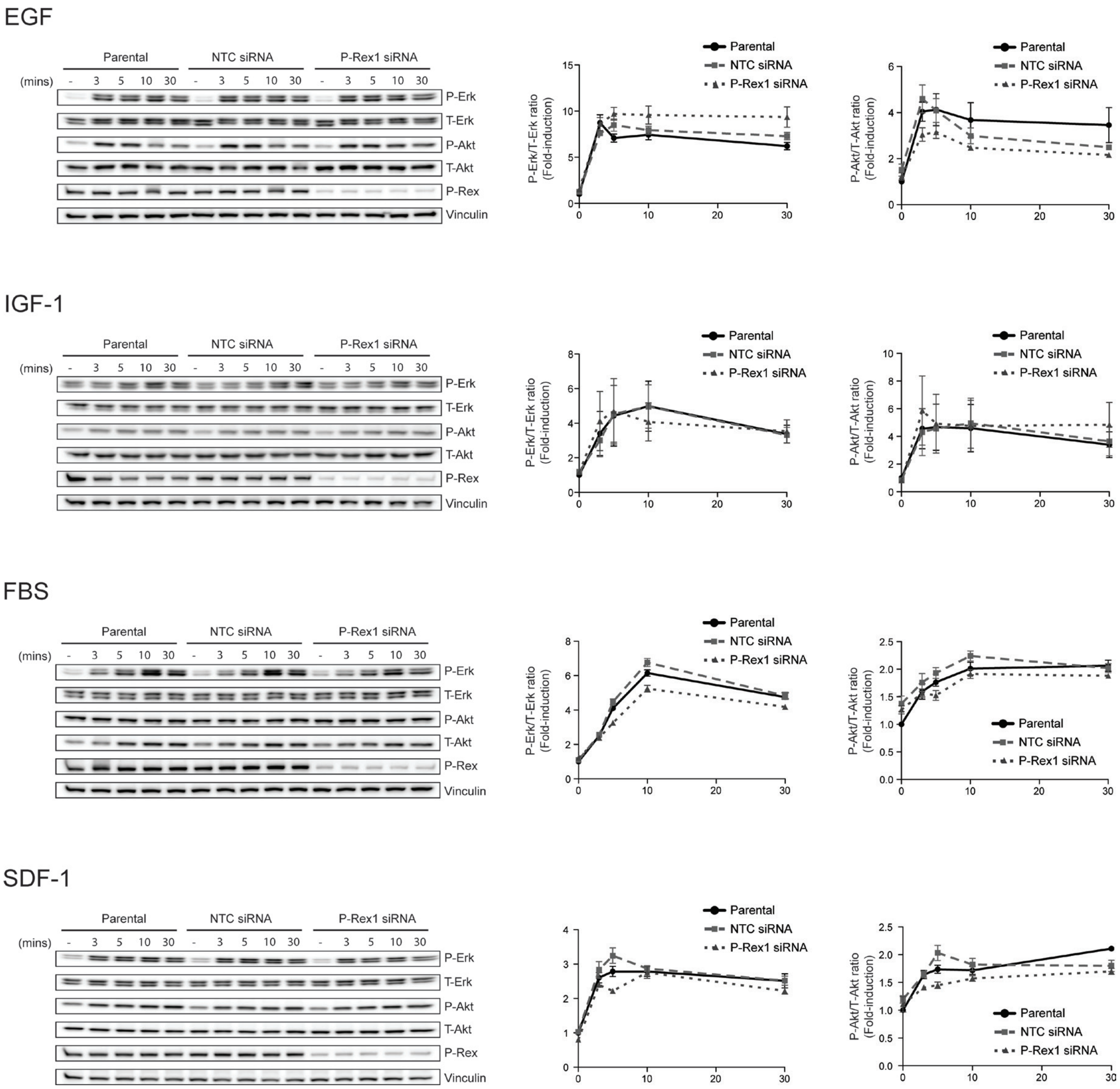

Supplementary Figure 1: Time-course of Erk and Akt activation in MCF-7 cells. Cells were transfected with P-Rex1 siRNA (pool) or non-target control (NTC) siRNA. After 16 h, cells were serum starved for 24 h and stimulated with EGF (100 ng/ml), IGF-I (100 ng/ml), FBS (10%), or SDF-1 (100 ng/ml) at different times. *Left panels*, representative experiments. *Middle and right panels*, densitometric analysis of phospho-Erk and phospho-Akt levels normalized to total levels. Results were expressed as fold-change relative to parental cells with vehicle stimulation. Data were expressed as mean ± S.E.M. of 3 independent experiments. No statistically significant differences were observed between parental, NTC and P-Rex1-depleted cells.

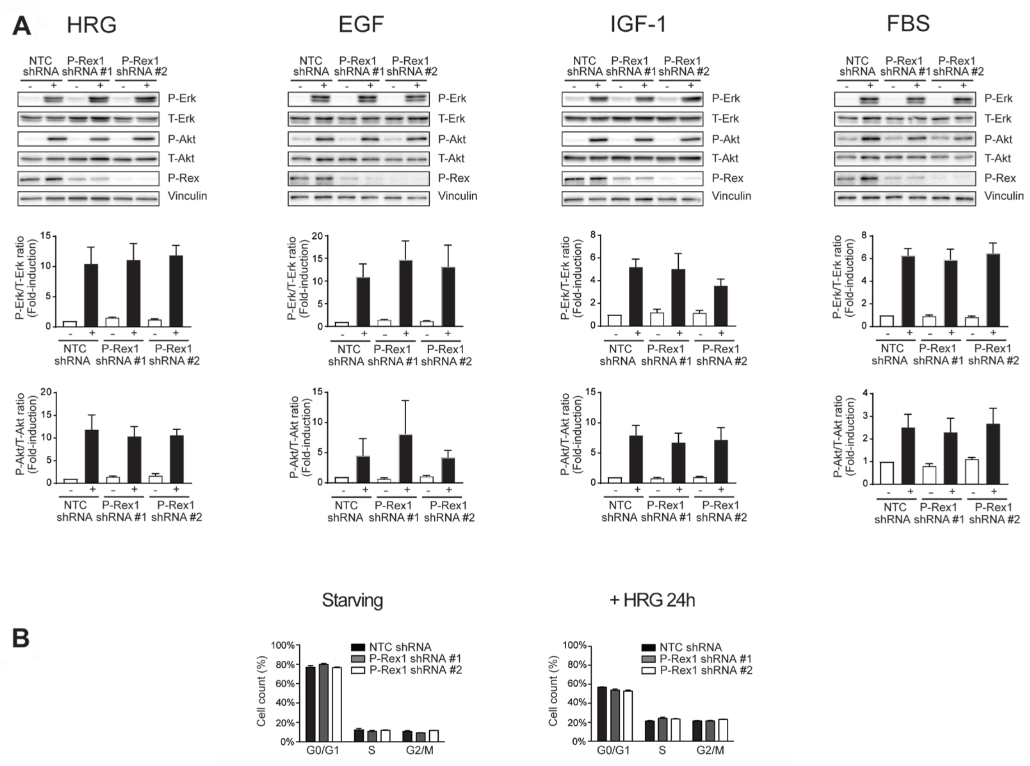

Supplementary Figure 2: Activation of Erk and Akt, and cell cycle analysis in MCF-7 cells subject to stable P-Rex1 silencing. P-Rex1-depleted MCF-7 cells lines were generated by infection with two different P-Rex1 shRNA lentiviruses (#1 and #2), followed by puromycin selection. As a control, a NTC shRNA lentivirus was used. (**A**) Cells were serum starved for 24 h and stimulated with HRG (20 ng/ml, 5 min), EGF (100 ng/ml, 2 min), IGF-I (100 ng/ml, 5 min) or FBS (10%, 10 min). Activation of Erk and Akt was determined by Western blot using phospho-specific antibodies. *Upper panels*, representative experiments. *Middle panels*, densitometric analysis of phospho-Erk, normalized to the total Erk. *Lower panels*, densitometric analysis of phospho-Akt, normalized to the total Akt. Results were expressed as fold-change relative to parental cells with vehicle stimulation. Data were expressed as mean ± S.E.M. of 3 independent experiments. (**B**) Cells were serum starved for 24 h, stimulated with HRG (20 ng/ml) or vehicle for 24 h, and subject to cell cycle analysis by FACS. Graphs show the distribution of cells in the different phases of cell cycle. Data are expressed as mean ± S.E.M. of 3 independent experiments.
